# Anti-integrin αvβ6 autoantibodies in patients with primary sclerosing cholangitis

**DOI:** 10.1007/s00535-023-02006-6

**Published:** 2023-06-13

**Authors:** Hiroyuki Yoshida, Masahiro Shiokawa, Takeshi Kuwada, Yuya Muramoto, Sakiko Ota, Yoshihiro Nishikawa, Hirona Maeda, Nobuyuki Kakiuchi, Kanako Okamoto, Hajime Yamazaki, Masataka Yokode, Takeharu Nakamura, Shimpei Matsumoto, Tomonori Hirano, Hirokazu Okada, Saiko Marui, Yuko Sogabe, Tomoaki Matsumori, Atsushi Mima, Norimitsu Uza, Yuji Eso, Atsushi Takai, Ken Takahashi, Yoshihide Ueda, Yuzo Kodama, Tsutomu Chiba, Hiroshi Seno

**Affiliations:** 1 Department of Gastroenterology and Hepatology, Kyoto University Graduate School of Medicine, Kyoto, Japan; 2 Division of Gastroenterology, Department of Internal Medicine, Kobe University Graduate School of Medicine, Hyogo, Japan; 3 Department of Pathology and Tumor Biology, Kyoto University, Kyoto, Japan; 4 Section of Clinical Epidemiology, Department of Community Medicine, Graduate School of Medicine, Kyoto University, Kyoto, Japan; 5 Kansai Electric Power Hospital, Osaka, Japan

**Keywords:** Biomarker, Inflammatory bowel diseases, Autoimmunity, Epithelial cell adhesion molecule, Fibronectin

## Abstract

**Background:**

Patients with primary sclerosing cholangitis (PSC) possess autoantibodies against biliary epithelial cells. However, the target molecules remain unknown.

**Methods:**

The sera of patients with PSC and controls were subjected to enzyme-linked immunosorbent assays to detect autoantibodies using recombinant integrin proteins. Integrin αvβ6 expression in the bile duct tissues was examined using immunofluorescence. The blocking activity of the autoantibodies was examined using solid-phase binding assays.

**Results:**

Anti-integrin αvβ6 antibodies were detected in 49/55 (89.1%) patients with PSC and 5/150 (3.3%) controls (*P* < 0.001), with a sensitivity and specificity of 89.1% and 96.7%, respectively, for PSC diagnosis. When focusing on the presence or absence of IBD, the proportion of the positive antibodies in PSC with IBD was 97.2% (35/36) and that in PSC alone was 73.7% (14/19) (*P* = 0.008). Integrin αvβ6 was expressed in bile duct epithelial cells. Immunoglobulin (Ig)G from 15/33 patients with PSC blocked integrin αvβ6-fibronectin binding through an RGD (Arg–Gly–Asp) tripeptide motif.

**Conclusions:**

Autoantibodies against integrin αvβ6 were detected in most patients with PSC; anti-integrin αvβ6 antibody may serve as a potential diagnostic biomarker for PSC.

**Supplementary Information:**

The online version contains supplementary material available at 10.1007/s00535-023-02006-6.

## Introduction

Primary sclerosing cholangitis (PSC) is an idiopathic and lethal cholestatic liver disease characterized by persistent and progressive biliary inflammation and fibrosis [1–4]. Although the cause and pathogenesis of PSC are unclear, it is generally accepted that both genetic and environmental factors contribute to the development of the disease. PSC diagnosis is based on cholangiographic findings, histology, and the absence of alternative diagnoses [5]. Various forms of secondary sclerosing cholangitis mimic PSC, thus complicating the diagnosis of PSC. Therefore, the development of specific diagnostic markers for PSC is needed.

Although multiple factors, such as genetic predisposition, environmental factors, dysbiosis, and dysregulated immune responses, are known to be involved in the pathogenesis of PSC, the exact underlying mechanisms remain unclear [1, 2]. The association with human leukocyte antigens and the presence of autoantibodies in the sera of patients with PSC support the important roles of immune-mediated mechanisms in PSC [1]. Patients with PSC have been reported to possess autoantibodies against biliary epithelial cells [6, 7]. Biliary epithelial cells are the main target of PSC. However, the target molecules or the mechanisms underlying the injuries have not been elucidated.

PSC is strongly associated with inflammatory bowel disease (IBD), suggesting common pathophysiological mechanisms between PSC and IBD [1]. We previously reported that anti-integrin αvβ6 autoantibodies are specifically found in patients with ulcerative colitis (UC), the most common type of IBD [8]. Recently, it was reported that most patients with UC in Europe and North America also possess anti-integrin αvβ6 autoantibodies [9, 10]. Integrins are a large family of heterodimeric cell surface receptors comprising two non-covalently associated α and β subunits that bind to the extracellular matrix (ECM) and mediate cell adhesion [11]. In mammals, 18 α and 8 β subunits have been identified that together form a minimum of 24 distinct heterodimers [11]. Among them, integrin αvβ6 is a receptor for ECM proteins, such as fibronectin [12], and its expression is restricted to epithelial cells [13]. Integrin αvβ6 on biliary epithelial cells is important for promoting the proliferation of biliary epithelial cells following liver injuries [14]. A recent report showed that germline mutations in human integrin β6, a subunit of integrin αvβ6, cause lethal cholestatic liver injuries and bloody diarrhea [15], which are major symptoms of PSC and UC, respectively, suggesting that integrin αvβ6 is a key molecule for PSC as well as UC.

Considering the close link between PSC and UC, the report that the human integrin β6 mutation causes PSC- and UC-like clinical characteristics [15], and the presence of anti-integrin αvβ6 autoantibodies in most patients with UC [8], we hypothesized that patients with PSC also possess autoantibodies against integrin family proteins, especially integrin αvβ6.

## Materials and methods

### Patients

We enrolled 55 patients with PSC and 150 controls [127 disease controls and 23 healthy controls (HCs)] in this study. The clinical characteristics of patients with PSC and the controls are summarized in Table 1 and Table S1. The patients with PSC were diagnosed based on serum biochemistry, cholangiogram, histological findings, and association with IBD. We also excluded secondary sclerosing cholangitis according to the method described by Ludiwig et al. [16]. The study did not include cases of small-duct PSC, and thus all the patients were categorized as large duct PSC, but included two cases of PSC-autoimmune hepatitis (AIH) overlap syndrome (PSC 41 and 46). Only five patients were treatment-naïve. The patients with UC were diagnosed according to a combination of symptoms, endoscopic findings, histologic findings, and the absence of alternative diagnoses [17, 18]. More detailed clinical information is provided in Table S2. It is generally considered that PSC-associated IBD and solitary UC are different diseases [19–21]. Indeed, colonic lesions of IBD accompanied by PSC are usually mild and mainly occur in the proximal colon. By contrast, solitary UC invariably affects the distal colon, including the rectum [21], and thus, IBD with PSC is often named PSC-associated IBD [22, 23]. Accordingly, in this study, we defined PSC, with or without IBD, as PSC and defined UC without PSC as UC.

**Table 1:** Clinical features of PSC patients and controls

	PSC with IBD (*n* = 36)	PSC without IBD (*n* = 20)	CCC (*n* = 32)	IgG4-SC (*n* = 14)	PBC (*n* = 39)	AIH (*n* = 15)	Collagen disease (*n* = 27)	Healthy control (*n* = 23)
Age								
Range	19–74	22–80	52–85	43–84	35–86	24–90	20–78	20–78
Median	39.0	42.5	70.0	70.0	66.0	69.0	56.0	56.0
Average	39.7	46.0	70.9	69.7	65.0	62.7	55.9	55.9
Sex male (%)	25 (69.4)	13 (65.0)	19 (59.4)	11 (78.6)	6 (15.4)	1 (6.7)	8 (29.6)	14 (60.9)
Age of diagnosis								
Range	11–66	18–76						
Median	27.0	39.0						
Average	30.1	41.0						
ALP^a^ (U/L)								
Range	57–1000	69–666						
Median	277.0	264.0						
Average	323.0	275.0						
PMS (%)								
0	31 (86.1)							
1	3 (8.3)							
2	1 (2.8)							
3	1 (2.8)							
> 4	0 (0)							
Rectal sparing (%)	16/25 (64)							

*PSC* primary sclerosing cholangitis, *IBD* inflammatory bowel disease, *CCC* cholangiocellular carcinoma, *IgG4-SC* IgG4-related sclerosing cholangitis, *PBC* primary biliary cholangitis, *AIH* autoimmune hepatitis, *ALP* alkaline phosphatase, *PMS* partial Mayo score

^a^The normal range of ALP is 38–113 U/L

In total, 36 of the 55 patients with PSC had IBD, and the remaining 19 had PSC alone. Among the 36 PSC patients with IBD, the partial Mayo score for IBD was 0 in 31 patients, 1 in 3 patients, 2 in 1 patient, and 3 in 1 patient (Table 1). Most patients with IBD and PSC were under remission. All the PSC patients underwent a colonoscopy to confirm the presence or absence of IBD. The diagnostic criteria for control diseases are listed in Table S3. There was no patient with UC among the disease controls. The serum samples of the study participants were obtained from January 2016 to February 2021 at the Kyoto University Hospital. The screening was performed on 17 patients with PSC and 12 controls; validation was performed with another 38 patients with PSC and 138 controls (Table S1). We used the sera of randomly selected 37 patients with PSC and 16 controls to examine IgG subclasses and antibody isotypes and performed a solid-phase integrin αvβ6 binding assay. All serum samples were stored at − 80 °C until assayed. Histologic analysis of frozen tissues was performed using bile duct tissues of 10 patients with PSC and 5 controls who underwent liver transplantation due to liver failure from other diseases or cholangiocarcinoma (Table S1).

The experiments were performed according to the Declaration of Helsinki and approved by the Ethics Committee of Kyoto University Graduate School and Faculty of Medicine (protocol number: R1004 and G738). All subjects provided written informed consent.

### ELISA

Integrins were screened using human recombinant proteins purchased from ACRO Biosystems (Newark, DE, USA) and R&D Systems (Minneapolis, MN, USA) (Table S4). For the detection of serum IgG antibodies against integrins, we used an ELISA Starter Accessory kit (E101, Bethyl Laboratories, Montgomery, TX, USA) following the manufacturer’s instructions. Briefly, microtiter plates were coated with 100 μL of 2 μg/mL recombinant proteins, incubated overnight at 4 °C, blocked, and incubated with 100 µL of diluted serum (1:100) or purified IgG (1:100) from patients for 60 min at room temperature. After washing, the plates were incubated with 100 µL of rabbit anti-human IgG antibody conjugated with horseradish peroxidase (HRP) (1:50,000; ab6759, Abcam, Cambridge, UK) at room temperature for 60 min. After washing, the bound reactants were detected by incubation with 3,3′,5,5′-tetramethylbenzidine for 7 min at room temperature. Absorbance was noted at 450 nm. Mg^2+^ and Ca^2+^ are important for integrin heterodimer formation and stability [24–26]. ELISA for integrin αvβ6 in the presence of Mg^2+^ and Ca^2+^ is effective in increasing the sensitivity and specificity [8]. Thus, in this study, all ELISAs were performed in the presence of MgCl_2_ and CaCl_2_ (1 mM each).

To examine the subclasses of the autoantibodies, we used the following secondary antibodies: anti-human IgG1, IgG2, IgG3, and IgG4 conjugated with HRP (1:2000; A-10648, Thermo Fisher Scientific Waltham, MA, USA; BS-AP007, BS-AP008, and BS-AP009; The Binding Site, Birmingham, UK). The following secondary antibodies were used to examine the isotypes of the autoantibodies: anti-human IgA, IgM, and IgE conjugated with HRP (1:50,000 A80-102P, 1:100,000 A80-100P, and 1:1000 A80-108P; Bethyl Laboratories).

To study whether the RGD (Arg–Gly–Asp) peptide inhibited the binding of patient IgG to integrin αvβ6, we added the RGDS (Arg–Gly–Asp–Ser) peptide (A9041, Sigma Aldrich, St. Louis, MO, USA) or the control RGES peptide (Arg–Gly–Glu–Ser) (A5686, Sigma Aldrich) to purified IgG before incubation.

### Preparation of human IgG

To purify IgG from the sera of patients with PSC and the controls, we used Ab-Rapid SPiN (P-013, ProteNova, Higashikagawa, Japan), according to the manufacturer’s instructions. The purified IgG was dialyzed against phosphate-buffered saline (PBS, pH 7.2), concentrated by ultrafiltration using an Amicon Ultra filter (UFC805024, Millipore, Darmstadt, Germany) to the same volume as the sera before purification, and stored at − 20 °C. The purified IgG concentration was measured using a Human IgG EIA kit (MK136, TaKaRa, Kusatsu, Japan). The purity of the IgG fraction was confirmed by testing for IgA, IgM, IgE, and protein contaminants using a Human IgA ELISA kit (E88-102, Bethyl Laboratories), a Human IgM ELISA kit (E88-100, Bethyl Laboratories), a Human IgE ELISA kit (E88-108, Bethyl Laboratories), and sodium dodecyl sulfate–polyacrylamide gel electrophoresis with Coomassie Brilliant Blue staining, respectively. The IgG recovery rate from the sera was confirmed to be > 90% in five patients with PSC and five controls, as in our previous study [27].

### Immunofluorescence

Immunofluorescence was performed according to standard methods for frozen tissues. The primary antibody was anti-integrin αvβ6 (1:1000; ab77906, Abcam), and the secondary antibody was Alexa Fluor 594 anti-mouse IgG (1:1000; A-11032, Thermo Fisher Scientific). All procedures were performed in the presence of MgCl_2_ and CaCl_2_ (1 mM each) because Mg^2+^ and Ca^2+^ are important for integrin heterodimer formation and stability, and the anti-integrin αvβ6 antibody detects the integrin αvβ6 heterodimer formation [8, 24].

### Solid-phase integrin αvβ6 binding assay

A solid-phase integrin αvβ6 binding assay was performed as previously described, with minor modifications [24]. Briefly, a 96-well microtiter plate was coated with 100 µL/well of 2 µg/mL integrin αvβ6 overnight at 4 °C, blocked, and then incubated with 100 µL of diluted patient or control IgG (1:10) for 60 min at room temperature. After washing five times with a wash solution, the plates were incubated with 100 µL of 2 µg/mL fibronectin (FC010, Millipore Sigma, Burlington, MA, USA) at room temperature for 60 min. After washing five times with a wash solution, an anti-fibronectin antibody (1:5000; ab2413, Abcam) was added, followed by incubation at room temperature for 60 min. After washing five times with a wash solution, an anti-rabbit IgG HRP-conjugated secondary antibody (1:10,000; A27036, Thermo Fisher Scientific) was added, followed by incubation at room temperature for 60 min. After washing five times with a wash solution, bound reactants were detected by incubation with 3,3′,5,5′-tetramethylbenzidine for 10 min at room temperature. Absorbance was determined at 450 nm. A solid-phase integrin αvβ6 binding assay was performed in the presence of MgCl_2_ and CaCl_2_ (1 mM each).

Before use, we determined that the anti-rabbit IgG HRP secondary antibody did not cross-react with the human IgG using an ELISA. Blank wells coated with integrin αvβ6 were incubated with fibronectin without patient or control IgG to calculate the inhibition rate. The inhibition rate was calculated as follows: (blank OD–sample OD)/blank OD. We used monoclonal anti-integrin αvβ6 antibody 10D5 (ab77906, Abcam) as a positive control.

### Statistical analysis

Statistical analyses were performed using GraphPad Prism version 9.1.2 (GraphPad, La Jolla, California, USA) or R version 3.6.3. Associations between categorical variables were tested using Fisher’s exact tests. The correlation between IgG antibody titers against integrin αvβ6 and the blocking activity of integrin αvβ6-fibronectin binding was evaluated using the Pearson product–moment correlation. Statistical significance was defined as *P* < 0.05.

## Results

### Detection of anti-integrin αvβ6 autoantibodies in sera of patients with PSC

**Fig. 1:**
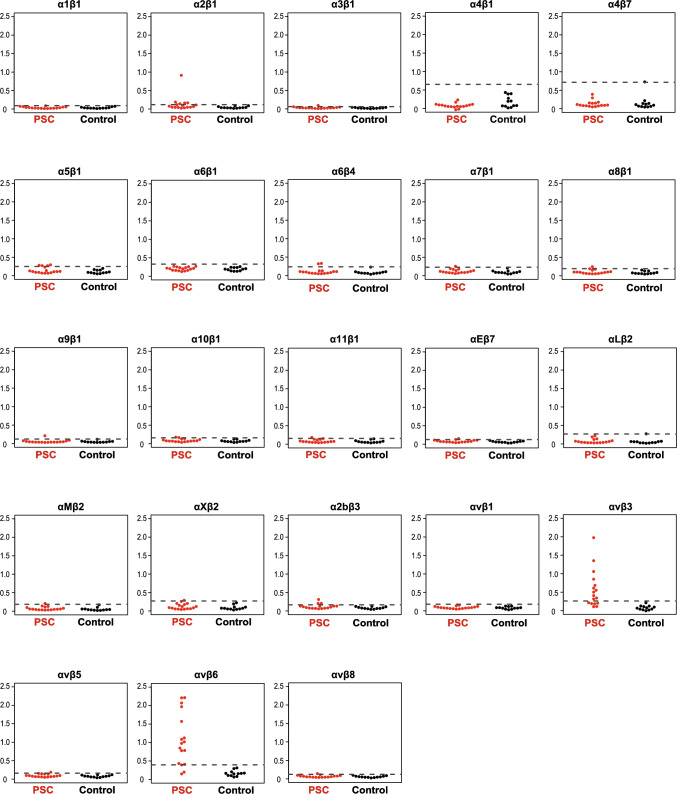
Screening of autoantibodies against various integrin proteins in sera from patients with PSC. Serum IgG antibodies against integrin family proteins were quantified using an enzyme-linked immunosorbent assay. Seventeen patients with PSC and 12 controls (2 CCC, 1 IgG4-SC, 3 AIH, 3 PBC patients, and 3 healthy controls; Table S1) were examined. The cut-off OD, defined as the mean plus three standard deviations of the control sera, is indicated by a dashed line. In total, 14 and 11 of the 17 PSC patients possessed IgG antibodies against integrin αvβ6 and αvβ3, respectively. Furthermore, 0–5 patients with PSC had IgG antibodies against other integrins. None or only 1 of the controls had IgG antibodies against any integrins. The y-axis shows the OD values of anti-integrin serum IgG levels (A450) against integrins. *PSC* primary sclerosing cholangitis, *IgG* immunoglobulin G, *CCC* cholangiocellular carcinoma, *IgG4-SC* IgG4-related sclerosing cholangitis, *AIH* autoimmune hepatitis, *PBC* primary biliary cholangitis, *OD* optical density

First, we examined whether the screening group of patients with PSC possessed autoantibodies against integrin family proteins. The sera of 17 patients with PSC and 12 controls (9 disease controls and 3 HCs; Table S1) were subjected to ELISAs for 23 recombinant integrin proteins (Fig. 1). We found that 15 (88.2%) and 11 (64.7%) patients with PSC possessed IgG antibodies against integrin αvβ6 and αvβ3, respectively. These values were based on a cutoff optical density (OD) of the mean plus three standard deviations of the control sera. By contrast, none or only a small number of patients with PSC possessed IgG antibodies against other integrins. Since integrin αvβ6 is exclusively expressed in epithelial cells including the bile duct [13], and the site of the injury in PSC is bile duct cells [1], and the sensitivity (88.2%) of the anti-integrin αvβ6 autoantibodies for PSC was the highest, we focused on integrin αvβ6 for further analyses.

**Fig. 2:**
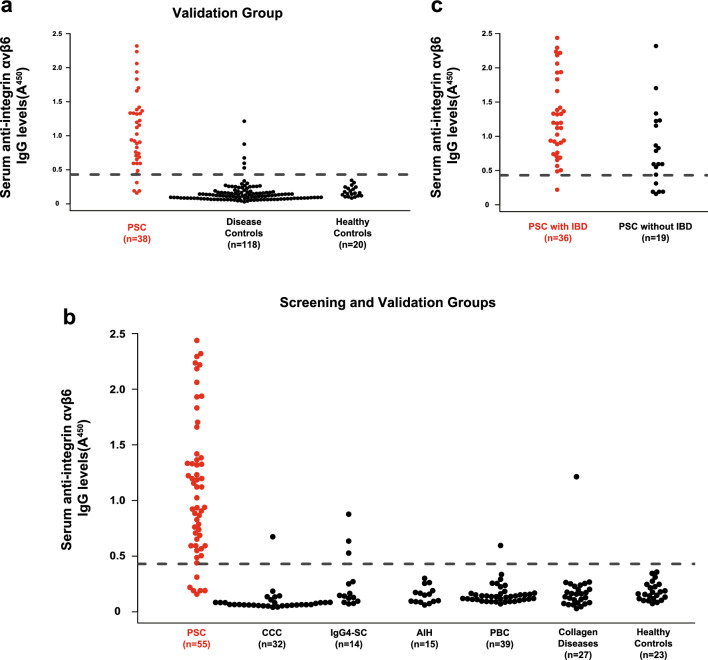
Detection of anti-integrin αvβ6 autoantibodies in sera from patients with PSC. Serum IgG antibodies against integrin αvβ6 were quantified by an ELISA. The cutoff OD, defined as the mean plus three standard deviations of the healthy control sera, is indicated by a dashed line. **a** A validation group comprising 38 patients with PSC, 118 disease controls, and 20 healthy controls (Table S1) was examined. **b** Patients and controls from the screening and validation groups were examined. **c** Patients with PSC with or without IBD were examined. *PSC* primary sclerosing cholangitis, *IgG* immunoglobulin G, *ELISA* enzyme-linked immunosorbent assay, *OD* optical density, *CCC* cholangiocellular carcinoma, *IgG4-SC* IgG4-related sclerosing cholangitis, *AIH* autoimmune hepatitis, *PBC* primary biliary cholangitis

In the screening group, the sensitivity and specificity of the anti-integrin αvβ6 IgG autoantibodies for PSC were 88.2% and 100%, respectively (Fig. 1). To validate the data of the screening group, we examined the sera of another 38 patients with PSC, 118 disease controls, and 20 HCs (Table S1). We found that 34/38 (89.5%) patients with PSC and 5/138 (3.6%) controls possessed IgG antibodies against integrin αvβ6 (*P* < 0.001) (Fig. 2a). When the screening and validation groups were combined, IgG antibodies against integrin αvβ6 were present in 49/55 (89.1%) patients with PSC and 5/150 (3.3%) controls (*P* < 0.001) (Fig. 2b). The sensitivity and specificity of the anti-integrin αvβ6 IgG autoantibodies for PSC were 89.1% and 96.7%, respectively. Furthermore, when focusing on the presence or absence of IBD, the proportion of the positive antibodies in PSC with associated IBD was 97.2% (35/36) and that in PSC alone was 73.7% (14/19) (*P* = 0.008) (Fig. 2c). Also, two of the two patients with PSC-AIH overlap syndrome had anti-integrin αvβ6 autoantibodies.

### Immunoglobulin G subclasses and isotypes of anti-integrin αvβ6 antibodies

**Fig. 3:**
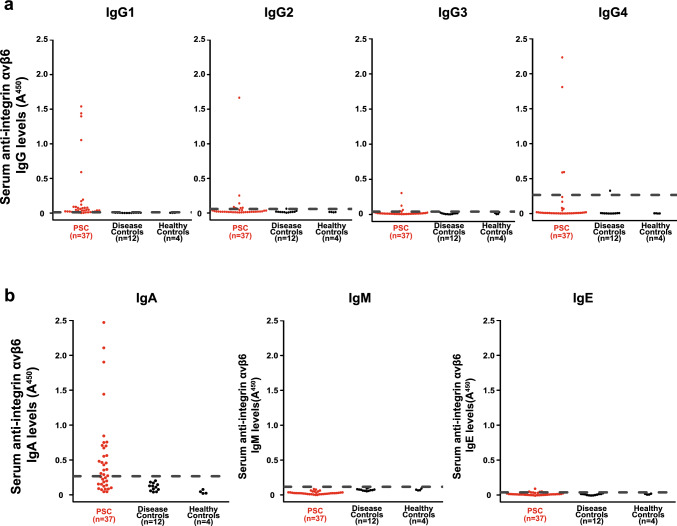
IgG subclasses and isotypes of the anti-integrin αvβ6 antibodies in sera from patients with PSC. **a** IgG subclasses of the anti-integrin αvβ6 antibodies were quantified by an ELISA. The cutoff OD, defined as the mean plus three standard deviations of the control sera, is indicated by a dashed line. Thirty (81.1%), 8 (21.6%), 5 (13.5%), and 4 (10.8%) of the 37 patients with PSC had IgG1, IgG2, IgG3, and IgG4 antibodies, respectively. Conversely, 1, 1, 0, and 1 of the 16 controls had IgG1, IgG2, IgG3, and IgG4 antibodies, respectively. **b** Isotypes of anti-integrin αvβ6 antibodies were quantified by ELISA. Twenty-one (56.8%), 0 (0%), and 4 (10.8%) of the 37 patients with PSC had IgA, IgM, and IgE antibodies against integrin αvβ6, respectively. Conversely, none of the 16 controls had IgA, IgM, or IgE antibodies. *IgG* immunoglobulin G, *PSC* primary sclerosing cholangitis, *ELISA* enzyme-linked immunosorbent assay, *OD* optical density

To further characterize the anti-integrin αvβ6 autoantibodies, we used the sera of randomly selected 37 patients with PSC and 16 controls (12 disease controls and 4 HCs). ELISA results showed that 30 (81.1%), 8 (21.6%), 5 (13.5%), and 4 (10.8%) of 37 patients with PSC possessed IgG1, IgG2, IgG3, and IgG4 antibodies, respectively (Fig. 3a). On the other hand, 21 (56.8%), 0 (0%), and 4 (10.8%) patients possessed IgA, IgM, and IgE antibodies, respectively (Fig. 3b). By contrast, controls had neither IgG1, IgG2, IgG3, IgA, IgM, nor IgE antibodies, and only one of the controls had an IgG4 antibody.

### Expression of integrin αvβ6 in bile duct epithelia

**Fig. 4:**
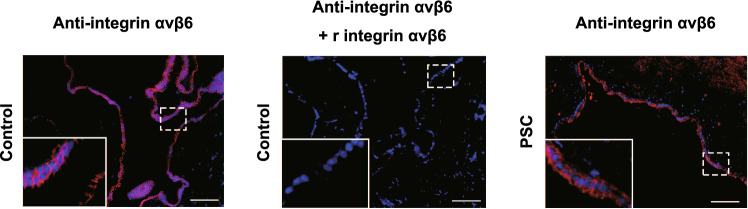
Immunofluorescence staining of integrin αvβ6 in human biliary tissue sections. Integrin αvβ6 was expressed on biliary epithelial layers of controls (left panel) and patients with PSC (right panel). A specific anti-integrin αvβ6 monoclonal antibody (10D5) [24] was used. The staining was abolished by preincubation with recombinant integrin αvβ6 (middle panel) but not other integrins (data not shown). Similar data were obtained from all patients (*n* = 10) and controls (*n* = 5) examined, and representative images are shown. The white boxes on the lower left are magnified images of the dashed line boxes. Scale bars: 100 μm. *PSC* primary sclerosing cholangitis

To examine the expression of integrin αvβ6 in the bile duct epithelium, we performed immunofluorescence staining using bile duct tissue samples from 10 patients with PSC and five disease controls (Table S1). We detected integrin αvβ6 in the bile duct epithelial cells in all patients with PSC and controls (Fig. 4, left and right panels). The staining was abolished by preincubation with recombinant integrin αvβ6 (Fig. 4, middle panel), suggesting that this reaction is specific.

### Blocking integrin αvβ6-fibronectin binding by IgG from patients with PSC

**Fig. 5:**
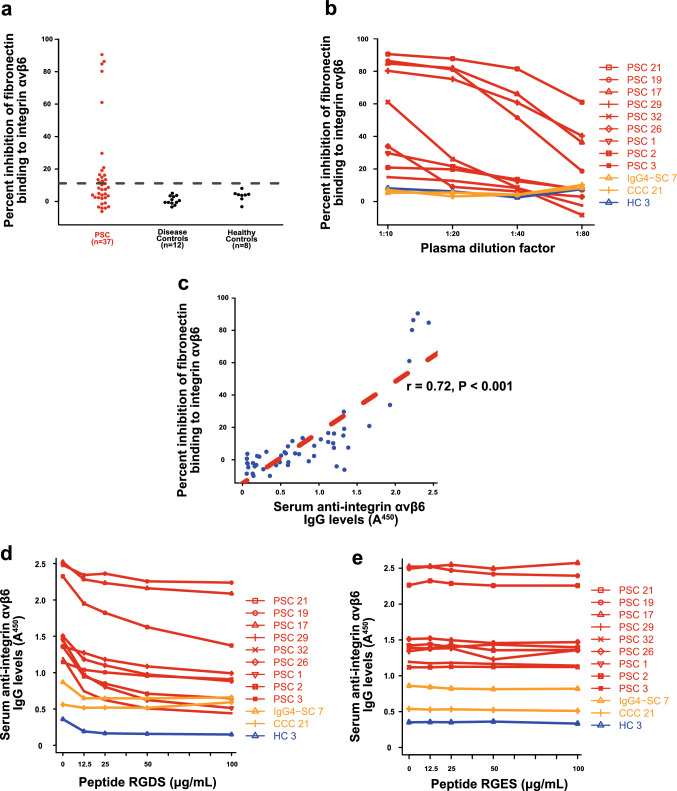
Blocking of integrin αvβ6-fibronectin binding by IgG of patients with PSC. **a** Inhibition of integrin αvβ6 binding to fibronectin by IgG from a patient with PSC was examined using a solid-phase binding assay. The cutoff OD, defined as the mean plus three standard deviations of the control IgG, is indicated by a dashed line. The assay showed that IgGs of 15/37 (40.5%) patients with PSC but none of the control IgGs blocked the binding of integrin αvβ6 to fibronectin. **b** Dose-dependent inhibition of binding of integrin αvβ6 to fibronectin by PSC patient IgG. IgGs of the patients with PSC with the anti-integrin αvβ6 antibody (PSC 21, 19, 17, 29, 32, 26, 1, 2, and 3) inhibited integrin αvβ6–fibronectin binding in a dose-dependent manner. Conversely, IgGs of controls with the anti-integrin αvβ6 antibody (IgG4-SC 7 and CCC 21) and a healthy control (HC 3) exhibited no blocking activity. **c** Titers of IgG antibodies against integrin αvβ6 were correlated with the blocking activity of integrin αvβ6-fibronectin binding (*r* = 0.72, *P* < 0.001). **d**, **e** Peptide RGDS (Arg–Gly–Asp–Ser) (**d**), but not RGES (Arg–Gly–Glu–Ser) (**e**), impaired binding of IgG from patients with PSC to integrin αvβ6 in a dose-dependent manner. We used the RGDS and RGES peptides to represent the RGD and RGE motifs, respectively [29]. *OD* optical density, *IgG* immunoglobulin G, *PSC* primary sclerosing cholangitis, *IgG4-SC* IgG4-related sclerosing cholangitis, *CCC* cholangiocellular carcinoma

In our previous study, anti-integrin αvβ6 autoantibody in patients with UC had an inhibitory effect on integrin αvβ6-fibronectin binding [8]. To investigate whether anti-integrin αvβ6 autoantibody in patients with PSC had the same effect, we conducted a solid-phase integrin αvβ6 binding assay (Fig. S1a). IgGs of 15/37 (40.5%) patients with PSC with anti-integrin αvβ6 autoantibody blocked integrin αvβ6-fibronectin binding (Fig. 5a); the monoclonal antibody 10D5 [24] was used as a positive control (Fig. S1b). Conversely, no control IgG exhibited the blocking activity (Fig. 5a). The blocking activity of patient IgG was dose-dependent (Fig. 5b) and correlated with the patient anti-integrin αvβ6 antibody titer (*r* = 0.72, *P* < 0.001; Fig. 5c).

Integrin αvβ6 binds to its ligands, such as fibronectin, by recognizing an RGD sequence in fibronectin [25]. Previously, we reported that anti-integrin αvβ6 antibodies in patients with UC and RGD peptides compete for binding to the integrin αvβ6, suggesting that anti-integrin αvβ6 antibodies in patients with UC exert their blocking effect by targeting the RGD-binding site of integrin αvβ6 [8]. We examined whether anti-integrin αvβ6 antibodies in patients with PSC also compete with RGD peptides. Results revealed that RGD peptides inhibited the binding of PSC patient IgG to integrin αvβ6 in a dose-dependent manner (Fig. 5d). By contrast, no such inhibitory effects were observed with the RGE peptide (control) (Fig. 5e). These findings are similar to those observed in UC [8]. However, the blocking activity of PSC patient IgG tended to be lower than that of UC patient IgG.

### Clinical relevance of the presence of anti-integrin αvβ6 antibodies

We compared the clinical characteristics of PSC patients with and without anti-integrin αvβ6 antibodies (Table 2). The frequency of presence of IBD was significantly higher in patients with anti-integrin αvβ6 antibodies than in those without. In addition, ALP, T-bil, and CRP tended to be higher in patients with anti-integrin αvβ6 antibodies than in those without, though not significant.

**Table 2:** Comparison of clinical characteristics between patients with anti-integrin αvβ6 antibody-positive and antibody-negative PSC

	Positive *n* = 49	Negative *n* = 6	*P* value
Age			0.48
Mean	40.8	45.3	
Median	40	50	
Sex			0.07
Male	36 (73.4%)	2 (33.3%)	
ALP^a^ U/L			0.17
Mean	321.5	190.7	
Median	277	232	
T-Bil^b^ mg/dl			0.30
Mean	4.8	1.4	
Median	1.7	0.9	
CRP^c^ mg/dl			0.22
Mean	1.5	0.4	
Median	0.8	0.1	
IBD			0.02
Presence	34 (69%)	1 (17%)	

*PSC* primary sclerosing cholangitis, *ALP* alkaline phosphatase, *T-Bil* total-bilirubin, *CRP* C-reactive protein, *IBD* inflammatory bowel disease

^a^The normal range of ALP is 38–113 U/L

^b^The normal range of T-Bil is 0.3–1.3 mg/dL

^c^The normal range of CRP is 0–0.2 mg/dL

We also examined whether presence of therapeutic intervention affected the positivity of anti-integrin αvβ6 antibodies. As a result, two out of three patients (67%) without medication and 46 out of 52 patients (88%) with medication had anti-integrin αvβ6 antibodies. There was no significant difference of the positivity of the autoantibody between PSC patients with and without medication (*P* = 0.29). On the other hand, 3/7 patients (43%) with endoscopic treatment of biliary tract and 46/48 patients (96%) without endoscopic treatment had anti-integrin αvβ6 antibodies. Thus, positivity of anti-integrin autoantibodies in patients with endoscopic treatment was significantly lower than that in patients without endoscopic treatment (*P* = 0.001).

## Discussion

This study revealed that most patients with PSC possessed anti-integrin αvβ6 autoantibodies. The sensitivity and specificity of anti-integrin αvβ6 antibodies for diagnosing PSC were very high. Immunofluorescence staining demonstrated the expression of integrin αvβ6 in biliary epithelial cells. In addition, IgG from half of the examined patients with PSC inhibited integrin αvβ6-fibronectin binding through the RGD motif.

Since there are no specific diagnostic markers for PSC, the diagnosis is based on non-specific biochemical, imaging, and pathological findings [1, 2, 5]. However, it is often difficult to differentiate PSC from other biliary diseases, such as cholangiocarcinoma, biliary-stone-associated cholangitis, and IgG4-related sclerosing cholangitis [5]. In addition, diagnostic imaging of endoscopic retrograde cholangiopancreatography carries a high risk of complications of acute pancreatitis. Here, we demonstrated that the anti-integrin αvβ6 antibody is a potential non-invasive diagnostic marker for PSC.

In this study, we found that integrin αvβ6 is expressed in biliary epithelial cells. Recently, Weil et al. reported that a patient with an integrin β6 germline mutation showed lethal cholestatic liver injuries and bloody diarrhea, characteristic clinical features of PSC and UC, respectively [15]. Moreover, Guillot et al. found that integrin αvβ6 is involved in biliary epithelial cell proliferation induced by bile acid-activated macrophages following liver injury in mice [14]. These data suggest that integrin αvβ6 plays an important role in the homeostasis of biliary epithelial cells. In this regard, it is noteworthy that IgGs from patients with PSC have inhibitory effects on integrin αvβ6-fibronectin binding. Thus, anti-integrin αvβ6 antibodies may play a pathological role in the development of PSC by disrupting the functions of integrin αvβ6 in biliary epithelial cells.

We recently reported that most patients with UC also possess anti-integrin αvβ6 antibodies [8]. Anti-integrin αvβ6 antibodies of patients with PSC or those with UC have similar characteristic features. The predominant subtypes of the antibodies of patients with both PSC and UC are IgG1; IgG of both patients inhibits integrin αvβ6-fibronectin binding via the RGD motif. Although integrin αvβ6 is present on the surface of both colonic [8] and biliary epithelial cells, the presence of anti-integrin αvβ6 antibodies in patients with both PSC and UC suggests common pathophysiological roles of the antibodies in PSC and UC; for example, disruption of integrin αvβ6-fibronectin binding results in epithelial cell-basement membrane dissociation. Nonetheless, although PSC is often associated with IBD, the colonic lesions in patients with PSC are different from those in patients with UC. For example, colonic lesions of IBD accompanied by PSC are usually mild and mainly occur in the proximal colon with rectal sparing and backwash ileitis. By contrast, UC invariably affects the distal colon, including the rectum, [21] and thus, IBD with PSC is often named PSC-associated IBD [22, 23]. Moreover, genome-wide association studies have demonstrated that a substantial number of single nucleotide polymorphisms of PSC are not shared by UC, and the presence of IBD in patients with PSC cannot be fully explained by shared genetic risk [28]. If anti-integrin αvβ6 antibodies play pathological roles in PSC or UC, the reason why those antibodies in patients with PSC and UC have different effects is unknown. The antigen epitope of the integrin αvβ6 recognized by anti-integrinαvβ6 antibodies of patients between PSC and UC may be different and induce different effects on different organs: the bile duct and colonic epithelium.

We observed in this study that prevalence of autoantibodies against integrin αvβ6 is different between PSC patients with and without IBD. The reason for this difference is unknown at present. In this regard, however, it may be noted that the titer of anti-integrin αvβ6 antibodies in PSC patients with IBD is higher than those without IBD (Fig. 2c). Therefore, one possibility is that some PSC patients with IBD who were negative for anti-integrin αvβ6 autoantibodies may have had very low levels of the antibody. Alternatively, some PSC patients without IBD may have other autoantibodies. However, further studies are needed to test these hypotheses.

One thing to be noted is that positivity of anti-integrin autoantibodies in patients who received endoscopic treatment was significantly lower than that in patients without endoscopic treatment. Because the sample size was small, whether the endoscopic treatment has an inhibitory role in the production of anti-integrin autoantibodies needs to be further clarified.

The present study has several limitations. The sample size was relatively small, and the study was limited to Japanese patients. The study outcomes warrant further investigations in patients of other ethnicities, with a large number of patients, to assess the wider application of these results.

Nevertheless, in conclusion, this study revealed for the first time that most patients with PSC possess autoantibodies against integrin αvβ6. These autoantibodies have a high specificity and sensitivity for PSC diagnosis. A more precise analysis of the characteristics of anti-integrin αvβ6 in patients with PSC and patients with UC is required.

## Supplementary Information

Below is the link to the electronic supplementary material.

Supplementary file1 (DOCX 154 KB)
