# Supplementary material for: Anti-integrin αvβ6 autoantibodies in patients with primary sclerosing cholangitis
Source: J Gastroenterol. 2023 Jun 13;58(8):778–89. doi: 10.1007/s00535-023-02006-6 (PMC10366314; doi:10.1007/s00535-023-02006-6)
Supplement: Supplementary file 1 — Supplementary file1 (DOCX 154 KB) [file 535_2023_2006_MOESM1_ESM.docx]

**Supplementary Fig. 1 Schematic representation of the solid-phase binding assay.**

**(a)** The binding assay was performed according to a method described previously, with minor modifications. Briefly, a 96-well microtiter plate was coated with integrin αvβ6, blocked, and incubated with patient or control IgG. Fibronectin, the anti-fibronectin primary antibody, and the anti-rabbit IgG HRP-conjugated secondary antibody were incubated with the antigen in series, with intermediate washing steps. The bound reactants were then detected using 3,3′,5,5′-tetramethylbenzidine. After coating with integrin αvβ6, Mg2+ and Ca2+ were added. Blank wells coated with integrin αvβ6 and incubated with fibronectin in the absence of patient or control IgG were used to calculate the inhibition rate as follows: (blank OD − sample OD) / blank OD. **(b)** Blocking of integrin αvβ6-fibronectin binding by monoclonal antibody 10D5 18 (positive control). The antibody inhibited integrin αvβ6-fibronectin binding in a dose-dependent manner. IgG, immunoglobulin G; HRP, horseradish peroxidase; OD, optical density

| **Supplementary Table 1. Clinical information for primary sclerosing cholangitis patients and controls related to Figure 1, 2, 3 and 5.** | | | | | | | | | | | | | |
| --- | --- | --- | --- | --- | --- | --- | --- | --- | --- | --- | --- | --- | --- |
| **PSC Patients** | | | | | | | | | | | | | |
| **Sample** | **Age** | **Sex** | **ALP ^a^ (U/L)** | **T-Bil ^b^ (mg/dL)** | **CRP ^c^ (mg/dL)** | **Diagnosis** | **Age of diagnosis** | **IBD presence or absence** | **Partial Mayo score** | **Screening group** | **Validation group** | **Study for subclasses, isotypes, and inhibitory activity** | **Study for liver tissues by immunofluorescence staining** |
|  |  |  |  |  |  |  |  |  |  |  |  |  |  |
|  |  |  |  |  |  |  |  |  |  |  |  |  |  |
| PSC1 | 32 | F | 834 | 1 | 0.1 | PSC | 15 | ○ | 0 |  | ○ | ○ |  |
| PSC2 | 38 | F | 317 | 6.5 | 0.6 | PSC | 38 | ○ | 0 |  | ○ | ○ |  |
| PSC3 | 47 | M | 445 | 1.2 | 2.1 | PSC | 33 | ○ | 0 |  | ○ | ○ |  |
| PSC4 | 51 | M | 558 | 4.4 | 0.8 | PSC | 35 | ○ | 0 |  | ○ | ○ |  |
| PSC5 | 42 | M | 69 | 0.9 | < 0.1 | PSC | 34 | × | - | ○ |  | ○ |  |
| PSC6 | 46 | M | 203 | 0.7 | 11.2 | PSC | 45 | ○ | 2 |  | ○ | ○ |  |
| PSC7 | 74 | M | 666 | 2.8 | 1.7 | PSC | 58 | ○ | 0 |  | ○ | ○ |  |
| PSC8 | 22 | F | 248 | 0.8 | 0.3 | PSC | 18 | × | - |  | ○ | ○ |  |
| PSC9 | 40 | M | 579 | 6.3 | 1 | PSC | 34 | ○ | - |  | ○ | ○ | ○ |
| PSC10 | 66 | F | 405 | 2.9 | 3.9 | PSC | 55 | × | - |  | ○ | ○ |  |
| PSC11 | 38 | M | 290 | 1.8 | 1 | PSC | 25 | ○ | 0 | ○ |  | ○ | ○ |
| PSC12 | 73 | M | 223 | 1.3 | 1.6 | PSC | 66 | ○ | 0 |  | ○ | ○ |  |
| PSC13 | 63 | M | 390 | 7.1 | 1.9 | PSC | 47 | ○ | 0 | ○ |  | ○ | ○ |
| PSC14 | 23 | M | 92 | 0.4 | < 0.1 | PSC | 20 | ○ | 0 |  | ○ | ○ |  |
| PSC15 | 43 | M | 307 | 0.8 | 0.4 | PSC | 42 | ○ | 1 |  | ○ | ○ |  |
| PSC16 | 43 | M | 666 | 1.7 | 1.2 | PSC | 37 | × | - |  | ○ | ○ |  |
| PSC17 | 45 | F | - | - | - | PSC | 22 | ○ | 0 | ○ |  | ○ |  |
| PSC18 | 29 | M | 217 | 23.7 | 1.3 | PSC | 26 | ○ | 1 |  | ○ | ○ | ○ |
| PSC19 | 34 | M | 63 | 0.7 | 0.2 | PSC | 17 | ○ | 0 |  | ○ | ○ |  |
| PSC20 | 47 | M | 264 | 8 | 3.8 | PSC | 37 | ○ | 0 | ○ |  | ○ |  |
| PSC21 | 35 | M | 207 | 1.8 | 0.3 | PSC | 28 | ○ | 0 | ○ |  | ○ |  |
| PSC22 | 45 | M | 341 | 19.7 | 4.3 | PSC | 32 | ○ | 0 |  | ○ | ○ | ○ |
| PSC23 | 29 | M | 202 | 1.8 | 0.5 | PSC | 11 | ○ | 0 | ○ |  | ○ |  |
| PSC24 | 38 | M | - | - | - | PSC | 34 | ○ | 0 | ○ |  | ○ |  |
| PSC25 | 28 | M | 87 | 0.5 | < 0.1 | PSC | 21 | ○ | 1 | ○ |  | ○ |  |
| PSC26 | 41 | M | 117 | 0.5 | < 0.1 | PSC | 41 | × | - | ○ |  | ○ |  |
| PSC27 | 53 | M | 647 | 8 | 1.1 | PSC | 38 | × | - | ○ |  | ○ | ○ |
| PSC28 | 25 | F | 57 | 0.9 | < 0.1 | PSC | 18 | ○ | 0 | ○ |  | ○ |  |
| PSC29 | 26 | F | 92 | 0.9 | 0.1 | PSC | 26 | ○ | 0 | ○ |  | ○ |  |
| PSC30 | 69 | F | 393 | 12.2 | 1.6 | PSC | 64 | × | - | ○ |  | ○ | ○ |
| PSC31 | 46 | F | 229 | 0.5 | 0.1 | PSC | 21 | ○ | 0 | ○ |  | ○ | ○ |
| PSC32 | 49 | F | 331 | 1.7 | < 0.1 | PSC | 30 | ○ | 0 | ○ |  | ○ |  |
| PSC33 | 63 | F | 271 | 3.3 | 1.7 | PSC | 44 | × | - |  | ○ | ○ |  |
| PSC34 | 55 | M | 290 | 5.7 | 1.4 | PSC | 26 | ○ | 0 |  | ○ | ○ | ○ |
| PSC35 | 46 | M | 105 | 1 | 0.2 | PSC | 46 | × | - |  | ○ | ○ |  |
| PSC36 | 35 | M | - | - | - | PSC | 23 | × | - |  | ○ | ○ |  |
| PSC37 | 29 | M | 88 | 0.9 | 4.8 | PSC | 26 | × | - | ○ | ○ | ○ | ○ |
| PSC38 | 23 | M | 440 | 6.4 | < 0.1 | PSC | 18 | × | - |  | ○ |  |  |
| PSC39 | 26 | M | 264 | 4.5 | < 0.1 | PSC | 23 | × | - |  | ○ |  |  |
| PSC40 | 34 | M | 1000 | 10.1 | 3.8 | PSC | 23 | ○ | 0 |  | ○ |  |  |
| PSC41 | 19 | M | 313 | 2.3 | 0.9 | PSC | 19 | ○ | 3 |  | ○ |  |  |
| PSC42 | 49 | M | 745 | 1.2 | 2.3 | PSC | 43 | ○ | 0 |  | ○ |  |  |
| PSC43 | 27 | M | 108 | 0,7 | 0.5 | PSC | 27 | × | - |  | ○ |  |  |
| PSC44 | 20 | M | 68 | 1.2 | 0.1 | PSC | 20 | ○ | 0 |  | ○ |  |  |
| PSC45 | 48 | F | 188 | 2.2 | 2.4 | PSC | 28 | ○ | 0 |  | ○ |  |  |
| PSC46 | 26 | F | 78 | 44.6 | 1.9 | PSC | 20 | ○ | 0 |  | ○ |  |  |
| PSC47 | 26 | M | 301 | 16.2 | 7.5 | PSC | 26 | × | - |  | ○ |  |  |
| PSC48 | 50 | F | 146 | 0.6 | 0.01 | PSC | 49 | ○ | 0 |  | ○ |  |  |
| PSC49 | 58 | F | 283 | 1.9 | 0.15 | PSC | 58 | × | - |  | ○ |  |  |
| PSC50 | 40 | M | 553 | 0.9 |  | PSC | 35 | × | - |  | ○ |  |  |
| PSC51 | 24 | F | 552 | 0.5 | 0.21 | PSC | 21 | ○ | 0 |  | ○ |  |  |
| PSC52 | 44 | M | 202 | 1.6 | 0.15 | PSC | 40 | × | - |  | ○ |  |  |
| PSC53 | 20 | M | 64 | 1.1 | 0.04 | PSC | 20 | ○ | 0 |  | ○ |  |  |
| PSC54 | 62 | M | 216 | 0.6 | 0.14 | PSC | 61 | × | - |  | ○ |  |  |
| PSC55 | 67 | F | 119 | 0.5 | 0.41 | PSC | 66 | × | - |  | ○ |  |  |
| **Disease Controls** | | | | | | | | | | | | | |
| CCC1 | 66 | M | 449 | 1.5 | 1.6 | CCC | 66 |  |  | ○ |  | ○ |  |
| CCC2 | 79 | M | 74 | 1.6 | 0.3 | CCC | 79 |  |  |  | ○ | ○ |  |
| CCC3 | 82 | M | 144 | 1.2 | 5.2 | CCC | 82 |  |  | ○ |  | ○ |  |
| CCC4 | 85 | F | 87 | 1.1 | 0.1 | CCC | 83 |  |  |  | ○ |  |  |
| CCC5 | 78 | M | 131 | 0.9 | 1.1 | CCC | 78 |  |  |  | ○ |  |  |
| CCC6 | 71 | M | 65 | 1 | 2 | CCC | 70 |  |  |  | ○ |  |  |
| CCC7 | 68 | F | 199 | 1.1 | 0.2 | CCC | 68 |  |  |  | ○ |  |  |
| CCC8 | 68 | F | 114 | 0.8 | < 0.1 | CCC | 68 |  |  |  | ○ |  |  |
| CCC9 | 71 | M | 119 | 2.4 | < 0.1 | CCC | 70 |  |  |  | ○ |  |  |
| CCC10 | 56 | M | 174 | 1.6 | 1.3 | CCC | 56 |  |  |  | ○ |  |  |
| CCC11 | 79 | F | 101 | 0.6 | 0.6 | CCC | 79 |  |  |  | ○ |  |  |
| CCC12 | 61 | F | 211 | 4.5 | 0.2 | CCC | 61 |  |  |  | ○ |  |  |
| CCC13 | 73 | M | 109 | 1.4 | 1.8 | CCC | 73 |  |  |  | ○ |  |  |
| CCC14 | 61 | F | 491 | 1.4 | 1.1 | CCC | 61 |  |  |  | ○ |  |  |
| CCC15 | 69 | F | 181 | 1 | < 0.1 | CCC | 67 |  |  |  | ○ |  |  |
| CCC16 | 73 | F | 244 | 1.7 | 2.5 | CCC | 73 |  |  |  | ○ |  |  |
| CCC17 | 77 | M | 95 | 0.9 | 1.6 | CCC | 77 |  |  |  | ○ |  |  |
| CCC18 | 76 | M | 291 | 0.6 | 0.3 | CCC | 76 |  |  |  | ○ |  |  |
| CCC19 | 73 | M | 155 | 0.5 | 0.9 | CCC | 70 |  |  |  | ○ |  |  |
| CCC20 | 69 | M | 85 | 1.5 | 0.2 | CCC | 69 |  |  |  | ○ |  |  |
| CCC21 | 66 | M | 279 | 9 | 0.4 | CCC | 66 |  |  |  | ○ |  |  |
| CCC22 | 85 | M | 96 | 0.7 | < 0.1 | CCC | 85 |  |  |  | ○ |  |  |
| CCC23 | 74 | M | 666 | 2.7 | 7 | CCC | 74 |  |  |  | ○ |  |  |
| CCC24 | 57 | F | 346 | 14.8 | 0.2 | CCC | 57 |  |  |  | ○ |  |  |
| CCC25 | 67 | M | 230 | 1.3 | 0.2 | CCC | 67 |  |  |  | ○ |  |  |
| CCC26 | 52 | F | 320 | 0.4 | 0.3 | CCC | 52 |  |  |  | ○ |  |  |
| CCC27 | 69 | M | 194 | 1 | 0.3 | CCC | 69 |  |  |  | ○ | ○ |  |
| CCC28 | 69 | F | 247 | 2.3 | 0.8 | CCC | 69 |  |  |  | ○ |  |  |
| CCC29 | 79 | M | 88 | 0.5 | 0.1 | CCC | 79 |  |  |  | ○ |  |  |
| CCC30 | 71 | M | 152 | 0.9 | < 0.1 | CCC | 71 |  |  |  | ○ |  |  |
| CCC31 | 83 | F | 229 | 0.6 | 2 | CCC | 83 |  |  |  | ○ |  |  |
| CCC32 | 71 | F | 134 | 0.9 | 2 | CCC | 71 |  |  |  | ○ |  |  |
|  | | | | | | | | | | | | | |
| IgG4-SC1 | 76 | M | 53 | 0.5 | 0.5 | IgG4-SC | 76 |  |  |  | ○ |  |  |
| IgG4-SC2 | 84 | M | 122 | 0.8 | 0.1 | IgG4-SC | 84 |  |  |  | ○ |  |  |
| IgG4-SC3 | 63 | M | 159 | 17.8 | 0.2 | IgG4-SC | 63 |  |  |  | ○ |  |  |
| IgG4-SC4 | 69 | M | 239 | 2.6 | 2.3 | IgG4-SC | 69 |  |  |  | ○ |  |  |
| IgG4-SC5 | 70 | F | 44 | 1 | 0.1 | IgG4-SC | 65 |  |  |  | ○ |  |  |
| IgG4-SC6 | 76 | M | 98 | 0.3 | 6.6 | IgG4-SC | 68 |  |  |  | ○ |  |  |
| IgG4-SC7 | 74 | M | 213 | 1.1 | 0.3 | IgG4-SC | 73 |  |  | ○ |  | ○ |  |
| IgG4-SC8 | 66 | M | 71 | 0.9 | 0.5 | IgG4-SC | 61 |  |  |  | ○ |  |  |
| IgG4-SC9 | 67 | F | 63 | 1.2 | 0.1 | IgG4-SC | 67 |  |  |  | ○ |  |  |
| IgG4-SC10 | 78 | M | 207 | 0.3 | 11.6 | IgG4-SC | 78 |  |  |  | ○ |  |  |
| IgG4-SC11 | 70 | M | 147 | 4.2 | 0.2 | IgG4-SC | 70 |  |  |  | ○ |  |  |
| IgG4-SC12 | 76 | F | 73 | 0.5 | 0.4 | IgG4-SC | 76 |  |  |  | ○ |  |  |
| IgG4-SC13 | 64 | M | 404 | 2.2 | 0.3 | IgG4-SC | 64 |  |  |  | ○ |  |  |
| IgG4-SC14 | 43 | M | 37 | 0.8 | < 0.1 | IgG4-SC | 41 |  |  |  | ○ |  |  |
|  | | | | | | | | | | | | | |
| PBC1 | 73 | F | 94 | 0.5 | 0.9 | PBC | 47 |  |  | ○ |  | ○ |  |
| PBC2 | 66 | F | 103 | 0.7 | < 0.1 | PBC | 56 |  |  | ○ |  | ○ |  |
| PBC3 | 43 | F | 74 | 0.3 | < 0.1 | PBC | 41 |  |  |  | ○ |  |  |
| PBC4 | 80 | F | 45 | 0.4 | < 0.1 | PBC | 68 |  |  | ○ |  | ○ |  |
| PBC5 | 54 | F | 93 | 1 | < 0.1 | PBC | 52 |  |  |  | ○ | ○ |  |
| PBC6 | 59 | F | 251 | 0.9 | 0.2 | PBC | 52 |  |  |  | ○ |  |  |
| PBC7 | 54 | F | 73 | 0.9 | < 0.1 | PBC | 47 |  |  |  | ○ |  |  |
| PBC8 | 63 | F | 139 | 1.2 | 0.1 | PBC | 48 |  |  |  | ○ |  |  |
| PBC9 | 75 | F | 52 | 0.6 | < 0.1 | PBC | 65 |  |  |  | ○ |  |  |
| PBC10 | 57 | M | 127 | 0.6 | 0.1 | PBC | 43 |  |  |  | ○ |  |  |
| PBC11 | 58 | F | 79 | 0.5 | < 0.1 | PBC | 43 |  |  |  | ○ |  |  |
| PBC12 | 71 | M | 96 | 0.7 | 0.5 | PBC | 60 |  |  |  | ○ |  |  |
| PBC13 | 70 | F | 92 | 0.4 | 0.2 | PBC | 57 |  |  |  | ○ |  |  |
| PBC14 | 76 | F | 51 | 0.4 | 0.4 | PBC | 69 |  |  |  | ○ |  |  |
| PBC15 | 56 | M | 222 | 7.2 | 1 | PBC | 48 |  |  |  | ○ |  | ○ |
| PBC16 | 59 | F | 194 | 0.7 | - | PBC | 52 |  |  |  | ○ |  |  |
| PBC17 | 70 | F | 73 | 1.6 | < 0.1 | PBC | 51 |  |  |  | ○ |  |  |
| PBC18 | 70 | F | 138 | 1.1 | - | PBC | 63 |  |  |  | ○ |  |  |
| PBC19 | 35 | F | 88 | 1.6 | 0.2 | PBC | 34 |  |  |  | ○ |  |  |
| PBC20 | 86 | M | 67 | 0.8 | 2 | PBC | 65 |  |  |  | ○ |  |  |
| PBC21 | 64 | F | 214 | 0.7 | 0.2 | PBC | 59 |  |  |  | ○ |  |  |
| PBC22 | 45 | F | 107 | 0.9 | < 0.1 | PBC | 35 |  |  |  | ○ |  |  |
| PBC23 | 78 | M | 228 | 0.6 | - | PBC | 70 |  |  |  | ○ |  |  |
| PBC24 | 55 | F | 162 | 0.6 | < 0.1 | PBC | 49 |  |  |  | ○ |  |  |
| PBC25 | 74 | F | 64 | 0.6 | <0.1 | PBC | 67 |  |  |  | ○ |  |  |
| PBC26 | 80 | F | 189 | 0.5 | 0.3 | PBC | 61 |  |  |  | ○ |  |  |
| PBC27 | 79 | F | 63 | 1 | < 0.1 | PBC | 56 |  |  |  | ○ |  |  |
| PBC28 | 53 | F | 224 | 0.7 | - | PBC | 47 |  |  |  | ○ |  |  |
| PBC29 | 80 | F | 196 | 0.5 | - | PBC | 63 |  |  |  | ○ |  |  |
| PBC30 | 73 | F | 68 | 0.3 | < 0.1 | PBC | 64 |  |  |  | ○ |  |  |
| PBC31 | 74 | F | 229 | 1.2 | - | PBC | 70 |  |  |  | ○ |  |  |
| PBC32 | 58 | F | 156 | 2.6 | 0.2 | PBC | 40 |  |  |  | ○ |  |  |
| PBC33 | 55 | F | 116 | 0.6 | < 0.1 | PBC | 38 |  |  |  | ○ |  |  |
| PBC34 | 73 | F | 100 | 0.8 | - | PBC | 65 |  |  |  | ○ |  |  |
| PBC35 | 75 | F | 103 | 0.7 | < 0.1 | PBC | 70 |  |  |  | ○ |  |  |
| PBC36 | 50 | F | 167 | 0.9 | 0.1 | PBC | 44 |  |  |  | ○ |  |  |
| PBC37 | 60 | M | 687 | 2.2 | - | PBC | 53 |  |  |  | ○ |  |  |
| PBC38 | 56 | F | 341 | 0.8 | - | PBC | 50 |  |  |  | ○ |  |  |
| PBC39 | 78 | F | 95 | 0.7 | 0.7 | PBC | 68 |  |  |  | ○ |  |  |
|  | | | | | | | | | | | | | |
| AIH1 | 80 | F | 56 | 3 | 0.4 | AIH | 80 |  |  |  | ○ |  |  |
| AIH2 | 48 | F | 205 | 0.2 | < 0.1 | AIH | 48 |  |  |  | ○ |  |  |
| AIH3 | 71 | F | 60 | 0.4 | 0.1 | AIH | 71 |  |  | ○ |  | ○ |  |
| AIH4 | 57 | F | 52 | 0.6 | 0.2 | AIH | 45 |  |  | ○ |  | ○ |  |
| AIH5 | 69 | F | 59 | 0.6 | 0.2 | AIH | 53 |  |  | ○ |  | ○ |  |
| AIH6 | 75 | F | 139 | 0.6 | < 0.1 | AIH | 54 |  |  |  | ○ |  |  |
| AIH7 | 69 | F | 47 | 1.1 | - | AIH | 63 |  |  |  | ○ |  |  |
| AIH8 | 73 | F | 47 | 1 | - | AIH | 53 |  |  |  | ○ |  |  |
| AIH9 | 29 | F | 100 | 0.5 | 0.2 | AIH | 22 |  |  |  | ○ |  |  |
| AIH10 | 72 | F | 18 | 0.9 | - | AIH | 64 |  |  |  | ○ |  |  |
| AIH11 | 24 | F | 49 | 0.4 | - | AIH | 12 |  |  |  | ○ |  |  |
| AIH12 | 55 | M | 76 | 1 | < 0.1 | AIH | 31 |  |  |  | ○ |  |  |
| AIH13 | 90 | F | 36 | 0.7 | 0.5 | AIH | 75 |  |  |  | ○ |  |  |
| AIH14 | 39 | F | 115 | 0.3 | 0.1 | AIH | 37 |  |  |  | ○ |  |  |
| AIH15 | 89 | F | 75 | 0.6 | - | AIH | 80 |  |  |  | ○ |  |  |
|  | | | | | | | | | | | | | |
| Collagen Diseases 1 | 73 | M |  |  |  | SSc |  |  |  |  | ○ |  |  |
| Collagen Diseases 2 | 78 | F |  |  |  | SSc |  |  |  |  | ○ |  |  |
| Collagen Diseases 3 | 55 | F |  |  |  | SSc |  |  |  |  | ○ |  |  |
| Collagen Diseases 4 | 54 | F |  |  |  | SSc |  |  |  |  | ○ |  |  |
| Collagen Diseases 5 | 61 | F |  |  |  | SSc |  |  |  |  | ○ |  |  |
| Collagen Diseases 6 | 77 | M |  |  |  | SSc |  |  |  |  | ○ |  |  |
| Collagen Diseases 7 | 46 | F |  |  |  | SSc |  |  |  |  | ○ |  |  |
| Collagen Diseases 8 | 49 | M |  |  |  | SSc |  |  |  |  | ○ |  |  |
| Collagen Diseases 9 | 58 | F |  |  |  | SSc |  |  |  |  | ○ |  |  |
| Collagen Diseases 10 | 44 | F |  |  |  | SSc |  |  |  |  | ○ |  |  |
| Collagen Diseases 11 | 56 | F |  |  |  | SLE |  |  |  |  | ○ |  |  |
| Collagen Diseases 12 | 20 | F |  |  |  | SLE |  |  |  |  | ○ |  |  |
| Collagen Diseases 13 | 78 | F |  |  |  | SLE |  |  |  |  | ○ |  |  |
| Collagen Diseases 14 | 20 | F |  |  |  | SLE |  |  |  |  | ○ |  |  |
| Collagen Diseases 15 | 32 | M |  |  |  | DM |  |  |  |  | ○ |  |  |
| Collagen Diseases 16 | 75 | M |  |  |  | DM |  |  |  |  | ○ |  |  |
| Collagen Diseases 17 | 56 | F |  |  |  | DM |  |  |  |  | ○ |  |  |
| Collagen Diseases 18 | 57 | F |  |  |  | CADM |  |  |  |  | ○ |  |  |
| Collagen Diseases 19 | 48 | F |  |  |  | CADM |  |  |  |  | ○ |  |  |
| Collagen Diseases 20 | 65 | M |  |  |  | PM |  |  |  |  | ○ |  |  |
| Collagen Diseases 21 | 68 | M |  |  |  | PM |  |  |  |  | ○ |  |  |
| Collagen Diseases 22 | 66 | F |  |  |  | DM |  |  |  |  | ○ |  |  |
| Collagen Diseases 23 | 47 | F |  |  |  | DM |  |  |  |  | ○ |  |  |
| Collagen Diseases 24 | 49 | F |  |  |  | CADM |  |  |  |  | ○ |  |  |
| Collagen Diseases 25 | 52 | F |  |  |  | DM |  |  |  |  | ○ |  |  |
| Collagen Diseases 26 | 73 | F |  |  |  | PM |  |  |  |  | ○ |  |  |
| Collagen Diseases 27 | 51 | M |  |  |  | PM |  |  |  |  | ○ |  |  |
|  | | | | | | | | | | | | | |
| Biliary atresia | 28 | M |  |  |  | Biliary atresia |  |  |  |  |  |  | ○ |
| Alcoholic cirrhosis | 56 | F |  |  |  | Alcoholic cirrhosis |  |  |  |  |  |  | ○ |
| Alcoholic cirrhosis | 56 | M |  |  |  | Alcoholic cirrhosis |  |  |  |  |  |  | ○ |
| Hepatitis B and C | 50 | F |  |  |  | Hepatitis B and C |  |  |  |  |  |  | ○ |
| **Healthy Controls** | | | | | | | | | | | | | |
| HC 1 | 73 | F |  |  |  | HC |  |  |  |  | ○ |  |  |
| HC 2 | 72 | M |  |  |  | HC |  |  |  |  | ○ |  |  |
| HC 3 | 84 | M |  |  |  | HC |  |  |  |  | ○ |  |  |
| HC 4 | 77 | F |  |  |  | HC |  |  |  |  | ○ |  |  |
| HC 5 | 79 | F |  |  |  | HC |  |  |  |  | ○ |  |  |
| HC 6 | 50 | M |  |  |  | HC |  |  |  |  | ○ |  |  |
| HC 7 | 77 | M |  |  |  | HC |  |  |  |  | ○ |  |  |
| HC 8 | 55 | F |  |  |  | HC |  |  |  |  | ○ |  |  |
| HC 9 | 70 | F |  |  |  | HC |  |  |  |  | ○ |  |  |
| HC 10 | 67 | F |  |  |  | HC |  |  |  |  | ○ |  |  |
| HC 11 | 73 | F |  |  |  | HC |  |  |  |  | ○ |  |  |
| HC 12 | 84 | M |  |  |  | HC |  |  |  |  | ○ |  |  |
| HC 13 | 42 | M |  |  |  | HC |  |  |  |  | ○ |  |  |
| HC 14 | 33 | M |  |  |  | HC |  |  |  | ○ |  | ○ |  |
| HC 15 | 33 | M |  |  |  | HC |  |  |  | ○ |  | ○ |  |
| HC 16 | 34 | M |  |  |  | HC |  |  |  | ○ |  | ○ |  |
| HC 17 | 35 | F |  |  |  | HC |  |  |  |  | ○ | ○ |  |
| HC 18 | 33 | M |  |  |  | HC |  |  |  |  | ○ |  |  |
| HC 19 | 36 | M |  |  |  | HC |  |  |  |  | ○ |  |  |
| HC 20 | 34 | F |  |  |  | HC |  |  |  |  | ○ |  |  |
| HC 21 | 34 | M |  |  |  | HC |  |  |  |  | ○ |  |  |
| HC 22 | 32 | M |  |  |  | HC |  |  |  |  | ○ |  |  |
| HC 23 | 34 | M |  |  |  | HC |  |  |  |  | ○ |  |  |

a The normal range of ALP is 38–113 U/L.

b The normal range of T-Bil is 0.3–1.3 mg/dL.

c The normal range of CRP is 0–0.2 mg/dL.

Abbreviations: PSC, primary sclerosing cholangitis; CCC, cholangiocellular carcinoma; IgG4-SC, IgG4-related sclerosing cholangitis; PBC, primary biliary cholangitis; AIH, autoimmune hepatitis; SLE, systemic lupus erythematosus; SSc, systemic sclerosis; CADM, clinically amyopathic dermatomyositis; PM, polymyositis; DM, dermatomyositis; HC, healthy control; IBD, inflammatory bowel disease; ALP, Alkaline phosphatase; T-Bil, Total-Bilirubin; CRP, C-reactive protein; M, male; F, female

| **Supplementary Table 2. More detailed clinical information on patients with PSC** | | | | | | | | | | |
| --- | --- | --- | --- | --- | --- | --- | --- | --- | --- | --- |
|  | **Medication** | **Anti-integrin αvβ6 antibody** | **AST ^a^ /ALT ^b^ (IU/L)** | **GGTP ^c^ (IU/L)** | **AIH presence or absence** | **IBD presence or absence** | **Colonic mucosa biopsy by colonoscopy** | **Intrahepatic bile duct disease** | **Extrahepatic bile duct disease** | **Endoscopic therapy for PSC** |
| **PSC1** | UDCA, 5-ASA | + | 293/314 | 1141 | - | + | + | + | - | - |
| **PSC2** | UDCA, 5-ASA, Rosuvastatin | + | 123/120 | 287 | - | + | + | + | + | - |
| **PSC3** | UDCA, 5-ASA, Camostat Mesilate, Famotidine | + | 79/91 | 338 | - | + | + | + | - | - |
| **PSC4** | UDCA, 5-ASA | + | 100/41 | 882 | - | + | + | + | + | Previous endoscopic biliary balloon dilation |
| **PSC5** | UDCA, Bezafibrate, Levofloxacin | - | 22/22 | 78 | - | - | + | + | + | Previous endoscopic biliary balloon dilation |
| **PSC6** | Esomeprazole, Trimebutine Maleate | + | 20/39 | 97 | - | + | + | + | - | - |
| **PSC7** | Aspirin, Lansoprazole, Hydroxycarbamide | + | 116/70 | 571 | - | + | + | + | + | - |
| **PSC8** | UDCA | - | 70/108 | 325 | - | - | + | + | + | Previous endoscopic biliary stenting |
| **PSC9** | - | + | 147/89 | 334 | - | + | + | + | + | - |
| **PSC10** | UDCA, Rosuvastatin | + | 90/94 | 249 | - | - | + | + | + | - |
| **PSC11** | UDCA, 5-ASA, Bezafibrate, Rabeprazole, Nalfurafine | + | 54/48 | 197 | - | + | + | + | + | - |
| **PSC12** | UDCA | + | 52/23 | 992 | - | + | + | + | - | Previous endoscopic biliary stenting |
| **PSC13** | UDCA, 5-ASA, Lansoprazole, Metronidazole | + | 121/53 | 216 | - | + | + | + | + | - |
| **PSC14** | UDCA, Salazosulfapyridine, Azathioprine | + | 27/35 | 125 | - | + | + | + | - | - |
| **PSC15** | UDCA, 5-ASA | + | 88/164 | 823 | - | + | + | + | + | - |
| **PSC16** | UDCA, PSL (2mg), Rabeprazole | + | 84/54 | 357 | - | - | + | + | - | - |
| **PSC17** | UDCA | + | - | - | - | + | + | + | + | - |
| **PSC18** | UDCA | + | 107/55 | 94 | - | + | + | + | - | - |
| **PSC19** | UDCA, 5-ASA, Lansoprazole | + | 14/6 _ | 19 | - | + | + | + | + | - |
| **PSC20** | UDCA, 5-ASA, Vonoprazan, Bezafibrate, Zinc acetate, | + | 122/41 | 100 | - | + | + | + | + | - |
| **PSC21** | UDCA, 5-ASA, Nalfurafine | + | 37/37 | 89 | - | + | + | + | + | - |
| **PSC22** | UDCA, 5-ASA, Rosuvastatin, Spironolactone | + | 131/92 | 119 | - | + | + | + | + | - |
| **PSC23** | UDCA | + | 56/68 | 197 | - | + | + | + | - | - |
| **PSC24** | UDCA, 5-ASA | + | - | - | - | + | + | + | + | - |
| **PSC25** | UDCA | + | 27/20 | 69 | - | + | + | + | + | Previous endoscopic biliary stenting |
| **PSC26** | UDCA | + | 14/19 | 181 | - | - | - | + | - | - |
| **PSC27** | UDCA, Zinc acetate | + | 190/205 | 423 | - | - | - | + | + | - |
| **PSC28** | UDCA, 5-ASA | - | 16/7 _ | 11 | - | + | + | + | + | - |
| **PSC29** | - | + | 55/50 | 143 | - | + | + | + | + | - |
| **PSC30** | Spironolactone, Furosemide | + | 89/63 | 97 | - | - | - | + | + | - |
| **PSC31** | UDCA, 5-ASA | + | 79/123 | 257 | - | + | + | + | + | - |
| **PSC32** | UDCA, Colestimide, Minocycline | + | 61/37 | 285 | - | + | + | + | - | - |
| **PSC33** | UDCA, Rifaximin, Salazosulfapyridine, Bucillamine, PSL (1.5mg) | - | 48/33 | 85 | - | - | - | + | + | Endoscopic biliary stent in the left lobe of the liver |
| **PSC34** | UDCA | + | 105/84 | 74 | - | + | - | + | + | - |
| **PSC35** | - | + | 16/20 | 51 | - | - | + | + | + | - |
| **PSC36** | UDCA, Esomeprazole, Amoxicillin, Clavulanate | + | - | - | - | - | + | + | + | - |
| **PSC37** | UDCA, Bezafibrate | + | 33/30 | 146 | - | - | - | + | - | - |
| **PSC38** | UDCA, Bezafibrate, Colestimide | + | 83/129 | 304 | - | - | + | + | + | - |
| **PSC39** | UDCA, Bezafibrate | + | 80/91 | 179 | - | - | + | + | - | - |
| **PSC40** | 5-ASA | + | 167/123 | 622 | - | + | + | + | - | - |
| **PSC41** | UDCA, Trimebutine maleate | + | 115/116 | 313 | + | + | + | + | - | - |
| **PSC42** | UDCA, 5-ASA, Lansoprazole, Ezetimibe | + | 80/52 | 745 | - | + | - | + | + | - |
| **PSC43** | UDCA | + | 21/29 | 108 | - | - | - | + | - | - |
| **PSC44** | 5-ASA | + | 13/21 | 183 | - | + | - | + | + | - |
| **PSC45** | UDCA, Nalfurafine, Cetirizine, Clemastine | + | 42/27 | 205 | - | + | + | + | + | - |
| **PSC46** | UDCA, PSL (5mg), Spironolactone, Furosemide, Tolvaptan, Rifaximin | + | 132/88 | 34 | + | + | + | + | - | - |
| **PSC47** | UDCA, Rifaximin, Spironolactone, Furosemide, Vonoprazan | + | 83/27 | 92 | - | - | + | + | + | - |
| **PSC48** | UDCA | + | 34/31 | 71 | - | + | + | + | - | - |
| **PSC49** | UDCA, Nalfurafine | - | 68/47 | 163 | - | - | - | + | - | Endoscopic biliary stent in place into both right and left lobes of the liver |
| **PSC50** | UDCA | + | 36/49 | 295 | - | - | - | + | - | - |
| **PSC51** | UDCA, 5-ASA, Rabeprazole | + | 137/241 | 659 | - | + | + | + | - | - |
| **PSC52** | UDCA | + | 24/30 | 57 | - | - | - | + | - | - |
| **PSC53** | - | + | 17/16 | 124 | - | + | + | + | - | - |
| **PSC54** | UDCA | - | 20/28 | 153 | - | - | - | + | - | - |
| **PSC55** | - | + | 20/39 | 209 | - | - | - | + | + | - |

a The normal range of AST is 13–30 IU/L.

b The normal range of ALT is 7–23 IU/L.

c The normal range of GGTP is 9–32 IU/L.

Abbreviations: PSC, primary sclerosing cholangitis; UDCA, Ursodeoxycholic Acid; 5-ASA, 5-Aminosalicylic Acid; PSL, Prednisolone; ALP, Alkaline phosphatase; T-Bil, Total-Bilirubin; CRP, C-reactive protein; AST, Aspartate aminotransferase; ALT, Alanine aminotransferase; GGTP, Gamma- -glutamyl transpeptidase; AIH, autoimmune hepatitis; IBD, inflammatory bowel disease

| **Supplementary Table 3. Related to Figure 1. Criteria for each control disease.** | |
| --- | --- |
| **Control Disease** | **Diagnosis** |
| autoimmune hepatitis | diagnosed by Autoimmune hepatitis: Diagnosis and treatment guide in Japan, 2013 (Hepatology Research 2014; 44: 368–370) |
| cholangiocellular carcinoma | Diagnosed by histology |
| Clinically amyopathic dermatomyositis | diagnosed by Japanese criteria for dermatomyositis and polymyositis (Mod Rheumatol. 2018; 28: 913-921) |
| Dermatomyositis | diagnosed by Japanese criteria for dermatomyositis and polymyositis (Mod Rheumatol. 2018; 28: 913-921) |
| IgG4-related sclerosing cholangitis | diagnosed by Clinical practice guidelines for IgG4-related sclerosing cholangitis (J Hepatobiliary Pancreat Sci. 2019; 26: 9-42.) |
| Lupus enteritis | diagnosed by 1997 American College of Rheumatology classification criteria in systemic lupus erythematosus |
| Polymyositis | diagnosed by Japanese criteria for dermatomyositis and polymyositis (Mod Rheumatol. 2018; 28: 913-921) |
| primary biliary cholangitis | diagnosed by Guidelines for the management of primary biliary cirrhosis (Hepatol Res. 2014 Jan;44 Suppl S1:71-90) |
| Systemic lupus erythematosus | diagnosed by 1997 American College of Rheumatology classification criteria in systemic lupus erythematosus (Arthritis Rheum. 1997; 40: 1725) |
| Systemic sclerosis | diagnosed by diagnostic criteria of systemic sclerosis (J Dermatol. 2018; 45: 633-691) |
| Ulcerative colitis | diagnosed by ulcerative colitis practice guidelines in adults (Am J Gatroenterol. 2010; 105: 501-523) |

| **Supplementary Table 4. Related to Figure 1 and S1. Antigens used for enzyme linked-immunosorbent assays.** | | | | |
| --- | --- | --- | --- | --- |
| **Protein name** | **Product code** | **Company** | **City** | **Country** |
| Recombinant Human Integrin α2bβ3 | IT3-H52W8 | ACROBiosystems | Newark | USA |
| Recombinant Human Integrin α1β1 | IT1-H52Wb | ACROBiosystems | Newark | USA |
| Recombinant Human Integrin α2β1 | IT1-H52W6 | ACROBiosystems | Newark | USA |
| Recombinant Human Integrin α3β1/VLA-3 | IT1-H52Wc | ACROBiosystems | Newark | USA |
| Recombinant Human Integrin α4β1 | IT1-H52W1 | ACROBiosystems | Newark | USA |
| Recombinant Human Integrin α4β7 | IT7-H82W9 | ACROBiosystems | Newark | USA |
| Recombinant Human Integrin α5β1 | IT1-H52W5 | ACROBiosystems | Newark | USA |
| Recombinant Human Integrin α6β1 | IT1-H52W7 | ACROBiosystems | Newark | USA |
| Recombinant Human Integrin α6(X1) β4 | 5497-A6 | R&D Systems | Minnesota | USA |
| Recombinant Human Integrin α7β1 | IT1-H52W8 | ACROBiosystems | Newark | USA |
| Recombinant Human Integrin α8β1 | IT1-H52W9 | ACROBiosystems | Newark | USA |
| Recombinant Human Integrin α9β1 | 5438-A9 | R&D Systems | Minnesota | USA |
| Recombinant Human Integrin α10β1 | IT1-H52Wa | ACROBiosystems | Newark | USA |
| Recombinant Human Integrin α11β1 | IT1-H52W3 | ACROBiosystems | Newark | USA |
| Recombinant Human Integrin αEβ7 | IT7-H52W7 | ACROBiosystems | Newark | USA |
| Recombinant Human Integrin αLβ2 | 3868-AV | R&D Systems | Minnesota | USA |
| Recombinant Human Integrin αMβ2 | IT2-H52W4 | ACROBiosystems | Newark | USA |
| Recombinant Human Integrin αVβ1 | IT1-H82W6 | ACROBiosystems | Newark | USA |
| Recombinant Human Integrin αVβ3 | IT3-H52E3 | ACROBiosystems | Newark | USA |
| Recombinant Human Integrin αVβ5 | IT5-H52W5 | ACROBiosystems | Newark | USA |
| Recombinant Human Integrin αVβ6 | IT6-H52E1 | ACROBiosystems | Newark | USA |
| Recombinant Human Integrin αVβ8 | IT8-H52W4 | ACROBiosystems | Newark | USA |
| Recombinant Human Integrin αXβ2 | 5755-AX | R&D Systems | Minnesota | USA |

| **Supplementary Table 5. all antibodies used in this study.** | | | | | |
| --- | --- | --- | --- | --- | --- |
| **Antibody name** | **Product code** | **Company** | **City** | **Country** | **RRID** |
| Rabbit Anti-Human IgG H&L (HRP) | ab6759 | Abcam | Cambridge | UK | AB_955434 |
| Mouse anti-Human IgG1 Fc Secondary Antibody, HRP | A-10648 | Thermo Fisher Scientific | Waltham | USA | AB_2534051 |
| Anti-IgG2 (Human) pAb-HRP | BS-AP007 | The Binding Site | Birmingham | UK |  |
| Anti-IgG3 (Human) pAb-HRP | BS-AP008 | The Binding Site | Birmingham | UK |  |
| Anti-IgG4 (Human) pAb-HRP | BS-AP009 | The Binding Site | Birmingham | UK |  |
| Goat anti-Human IgA Antibody HRP Conjugated | A80-102P | Bethyl Laboratories | Montgomery | USA | AB_67047 |
| Goat anti-Human IgM Antibody HRP Conjugated | A80-100P | Bethyl Laboratories | Montgomery | USA | AB_67082 |
| Goat anti-Human IgE Antibody HRP Conjugated | A80-108P | Bethyl Laboratories | Montgomery | USA | AB_67056 |
| Mouse monoclonal [10D5] to Integrin alpha V + beta 6 | ab77906 | Abcam | Cambridge | UK | AB_1603055 |
| Goat anti-Mouse IgG (H+L) Highly Cross-Adsorbed Secondary Antibody, Alexa Fluor™ 594 | A-11032 | Thermo Fisher Scientific | Waltham | USA | AB_2534091 |
| Anti-Fibronectin antibody | ab2413 | Abcam | Cambridge | UK | AB_2262874 |
| Goat anti-Rabbit IgG (Heavy Chain), Superclonal™ Recombinant Secondary Antibody, HRP | A27036 | Thermo Fisher Scientific | Waltham | USA | AB_2536099 |
| Anti-Integrin alpha V antibody [EPR16800] | ab179475 | Abcam | Cambridge | UK | AB_2716738 |
| Anti-ITGB6 antibody produced in rabbit | HPA023626 | Sigma Aldrich | St. Louis | USA | AB_2671267 |
| Mouse IgG HRP Linked Whole Ab | NA931 | Cytiva | Tokyo | Japan | AB_772210 |
| Donkey anti-Rabbit IgG (H+L) Cross-Adsorbed Secondary Antibody, HRP | 31458 | Thermo Fisher Scientific | Waltham | USA | AB_228213 |
